# EVALUATING A CO-DESIGNED EXERGAMING PLATFORM (MOUVMAT) FOR OLDER ADULTS LIVING IN LONG-TERM CARE HOMES

**DOI:** 10.1093/geroni/igad104.1747

**Published:** 2023-12-21

**Authors:** Charlene Chu, Renée Biss, Haniya Bharucha, Henrique Matulis

**Affiliations:** University of Toronto, Toronto, Ontario, Canada; University of Windsor, Windsor, Ontario, Canada; University of Toronto, Toronto, Ontario, Canada; University of Toronto, Toronto, Ontario, Canada

## Abstract

MouvMat is an exergaming technology with an interactive digital gaming surface that was co-designed with a user-centered design approach for older adults (OA) living in long term care homes. The purpose of the study was to evaluate the acceptability and efficacy of MouvMat to improve mobility, cognitive function and psychosocial wellbeing among OAs. The study was conducted as a multi-site pilot randomised controlled trial where OAs in the intervention group attended 1-hour group sessions 3 times per week for 6 weeks. The control group received standard recreational programing. All participants underwent assessments (TUG, 2MW test, UCLA Loneliness Scale, Cornell Scale of Depression, digit span, TMT-A, TMT-B, alternating sequences, and verbal fluency) at baseline, mid-intervention, and end of intervention. Participant ratings indicated that the MouvMat was acceptable and OAs with a range of comorbid health conditions were able to attend sessions and engage with the intervention. Efficacy findings were varied and should be interpreted in the context of the heterogeneous physical and cognitive abilities of participants living in a long-term care setting. Future studies will focus on larger sample size and improvement in MouvMat’s design and interface.

